# TRANSFORMATIVE CARE: LIFESTYLE INTERVENTIONS IN UNDERSERVED AGING

**DOI:** 10.1093/geroni/igae098.2644

**Published:** 2024-12-31

**Authors:** Kristen Neises

**Affiliations:** University of Louisville, Louisville, Kentucky, United States

## Abstract

In response to the escalating challenges posed by an increasingly aging population in the United States, particularly in rural areas, an urgent demand arises for innovative healthcare solutions. This presentation illuminates the formidable hurdles faced by older adults with multiple chronic conditions, most of which result from lifestyle. The Optimal Aging Lifestyle Medicine (OALM) program emerges as a catalyst for transformative change in older adult care. Focused on medically underserved regions, this program empowers healthcare professionals through education in age and dementia-friendly lifestyle medicine, emphasizing evidence-based lifestyle interventions for preventing, managing, and reversing chronic diseases. The OALM program forges a collaborative care approach, uniting professionals across disciplines using the Flourish Model and extending outreach via Project ECHO to remote rural areas. Its mission encompasses filling training gaps by educating healthcare workers across Kentucky and evolving a traditional geriatric clinic into an innovative lifestyle medicine center for older adults. This presentation delves into the integration of lifestyle medicine into geriatric clinics, explores older adults’ perspectives on care through focus group discussions, and the development of an interdisciplinary lifestyle medicine curriculum. Case studies will spotlight a resilient patient care approach, highlighting successes and addressing challenges in groundbreaking initiatives navigating the intricacies of geriatric care, ultimately optimizing aging outcomes and reshaping narratives in rural communities.

